# Quantitative Evaluation of Nucleic Acid Degradability of Copper Alloy Surfaces and Its Correlation to Antibacterial Activity

**DOI:** 10.3390/antibiotics10121439

**Published:** 2021-11-24

**Authors:** Akiko Yamamoto, Shinji Tanaka, Keiichiro Ohishi

**Affiliations:** 1 Research Center for Functional Materials, National Institute for Materials Science, 1-1 Namiki, Tsukuba 305-0044, Japan; 2 Research & Development Department, Sambo Plant, Mitsubishi Materials Corporation, Sambo-cho 8-374, Sakai-ku, Sakai-shi 590-0906, Japan; tanakas@mmc.co.jp (S.T.); oishik@mmc.co.jp (K.O.)

**Keywords:** antimicrobial activities, antibacterial test, copper and copper alloys, DNA degradability, a swab method

## Abstract

Copper (Cu) and its alloys have bactericidal activity known as “contact killing” with degradation of nucleic acids inside the bacteria, which is beneficial to inhibit horizontal gene transfer (HGF). In order to understand the nucleic acid degradability of Cu and its alloy surfaces, we developed a new in vitro method to quantitatively evaluate it by a swab method under a “dry” condition and compared it with that of commercially available antibacterial materials such as antibacterial stainless steel, pure silver, and antibacterial resins. As a result, only Cu and its alloys showed continuous degradation of nucleic acids for up to 6 h of contact time. The nucleic acid degradability levels of the Cu alloys and other antibacterial materials correlate to their antibacterial activities evaluated by a film method referring to JIS Z 2801:2012 for Gram-negative (*Escherichia coli*) and Gram-positive (*Staphylococcus aureus*) bacteria. Nucleic acid degradation by copper (I) and (II) chlorides was confirmed at the ranges over 10 mM and 1–20 mM, respectively, suggesting that the copper ion release may be responsible for the degradation of the nucleic acids on Cu and its alloy surfaces. In conclusion, the higher Cu content in the alloys gave higher nucleic acid degradability and higher antibacterial activities.

## 1. Introduction

Antimicrobial resistance (AMR) in bacteria has become a global threat, and deaths attributable to AMR are estimated to reach over 10 million people by 2050 [1]. Unnecessary and excessive antimicrobial use should be avoided worldwide, suggesting the necessity of new sanitation methods without antimicrobials. Contaminated touch surfaces in hospital rooms play an important role in the transmission of healthcare-associated pathogens [2], thus, it is important to control the number of viable bacteria (bioburden) on touch surfaces, since methicillin-resistant *Staphylococcus aureus* (MRSA), one of the causes of healthcare-associated infection (HAI) in hospitals, can survive for months with infectivity on general touch surfaces of resin and stainless steel [3]. Under these circumstances, metal copper and its alloys have attracted attention in healthcare-related fields due to their excellent antimicrobial activity. Much research has been carried out to conclude that microorganisms, including Gram-negative and Gram-positive bacteria, fungi, spores, yeasts, and viruses, are rapidly killed on copper and its alloy surfaces, which is described by the term “contact killing” [4,5,6]. Prolonged exposure of microorganisms to copper and its alloy surfaces resulted in no recovery of viable microorganisms. Therefore, copper and its alloys containing copper with over 60% in their composition were registered at the US Environmental Protection Agency as the first solid antimicrobial material [6]. Introduction of copper and its alloys to the touch surfaces such as door handles, washroom fixtures, and bed rails have been carried out in hospitals and other healthcare-related institutes to achieve the reduction of bioburden and HAI [7,8,9,10]. Generally, higher antibacterial activity is obtained with copper alloys having higher copper contents [4,11]. However, pure copper easily changes its surface color by oxidation; therefore, copper alloys are practical candidates to apply touch surfaces.

Another feature of the antibacterial activity of copper and its alloys is that only a few copper-resistant bacteria have been reported. This may be related to the degradation of bacterial nucleic acids observed on copper and its alloy surfaces [12,13,14,15,16,17,18]. Bacteria have the ability of horizontal gene transfer (HGT), which can occur on touch surfaces. By HGT, the antibiotic-resistant gene can be transferred to another bacterium that never encounters a corresponding antibiotic; this process may contribute to the generation of multi-antibiotic-resistant bacteria [15]. HGT can occur even with remaining nucleic acids from dead bacteria, therefore, the degradability of bacterial nucleic acids on the material surface is one of the essential properties for the materials applied to these touch surfaces. For its further utilization and optimization, it is important to appropriately evaluate the nucleic acids degradability of materials as well as to investigate its mechanism. In order to evaluate the fragmentation of bacterial genes, electrophoresis of nucleic acids extracted from the bacteria applied onto the material surface is mostly performed [12,14,15,16,17,18], but a quantitative evaluation is difficult since bacterial numbers will change at the material surface depending on its antibacterial activity. There is no quantitative evaluation method of a material’s ability to degrade nucleic acids on its surface.

In this study, we developed a new in vitro method to evaluate the nucleic acid degradability of the material surface. Pure copper, three kinds of copper alloys (CLEANBRASS^®^, CLEANBRIGHT^®^, and C6932), and commercially available antibacterial materials such as resins (X and Y) and an antibacterial stainless steel (NSSAM3 [19], abbreviated as ABSS) were employed. Pure silver (Ag) is also tested as a reference material. Nucleic acid degradability of these material surfaces was evaluated and its correlation to their antibacterial activity for Gram-negative (*Escherichia coli*) and Gram-positive (*Staphylococcus aureus*) bacteria was examined.

## 2. Results

### 2.1. Nucleic Acid Degradability of Testing Materials

In our developed method, a 1 µL portion of nucleic acid solution was directly spread onto the material surface, which dries within 5 min, simulating the situation of an airborne droplet. After a certain period of contact time, the material surface was wiped with a polyester swab wetted by a small portion (20 µL) of an extract solvent (0.03% sodium dodecyl sulphate; SDS). Then, the remaining nucleic acid in the swab was further extracted into the solvent and quantified using a fluorescent die, acridine orange (AO), a nucleic acid intercalater. Since the bacterial cells are not involved, the nucleic acid degradability of the material can be evaluated independently from the bacterial growth or death on its surface. This kind of swabbing method is widely employed to monitor bioburden in a hospital environment [8], but not for the evaluation of nucleic acid degradability. A non-ionic surfactant was added to the nucleic acid solution, enabling us to control its spreadability and drying time on the different material surfaces.

**Figure 1:**
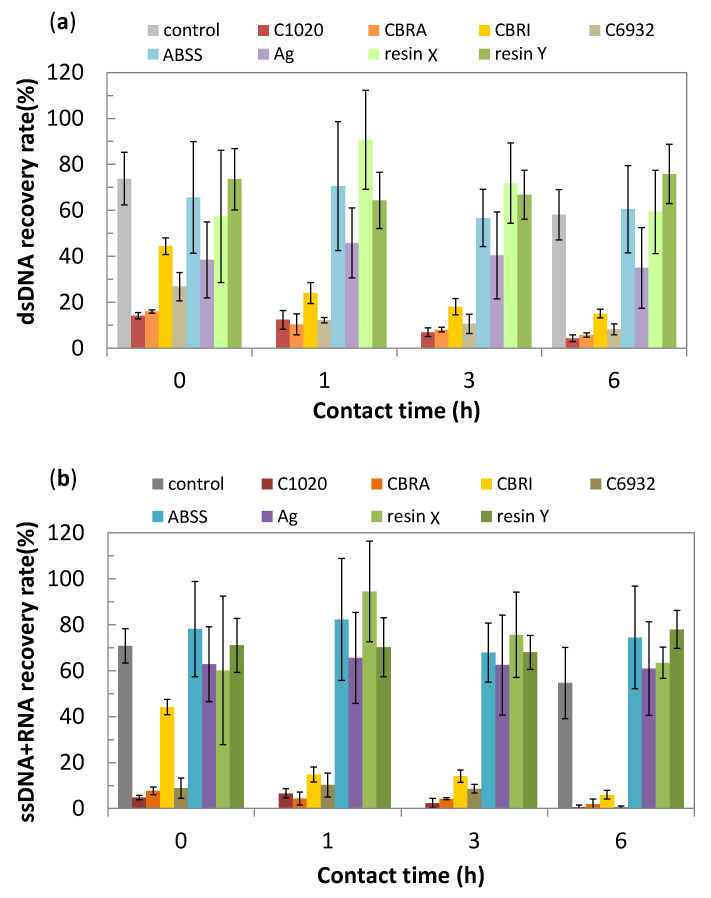
Recovery rates of (**a**) double-stranded deoxyribonucleic acids (dsDNAs) and (**b**) single-stranded (ss) DNA and ribonucleic acid (RNA) contacted on the material surface after a certain period of time (mean ± standard deviation).

The results of nucleic acid degradation assay are shown in Figure 1. The average recovery rates of nucleic acids from the glass surface were 70–75% at time “0” (5 min), which suggests the loss of the nucleic acid samples due to swabbing and extracting processes in this assay. However, the recovery rates from the pure copper (C1020) were 14.2% for double-stranded deoxyribonucleic acids (dsDNA) and 4.8% for single-stranded DNA and ribonucleic acids (ssDNA + RNA) even at time “0”. The recovery rates from copper alloys were 8–27%, except CLEANBRIGHT^®^ (CBRI). The recovery rates from CBRI were 44% for both of dsDNA and ssDNA + RNA at time “0”, but they decreased to 15.1 and 6.0%, respectively, with an increase in contact time. In the cases of ABSS, Ag, and resins, the recovery rates of nucleic acids stayed 40–75% regardless of their contact time. The metallic silver had a relatively low recovery rate of dsDNA as ~40% than other non-copper materials, which was closer to that of CBRI, but it was stable through the contact time. The recovery rate of nucleic acids may be influenced by their adsorption kinetics to different material surfaces, therefore, the time-dependent decrease in the recovery rate is considered as a good indicator of the material’s nucleic acids degradability.

**Figure 2:**
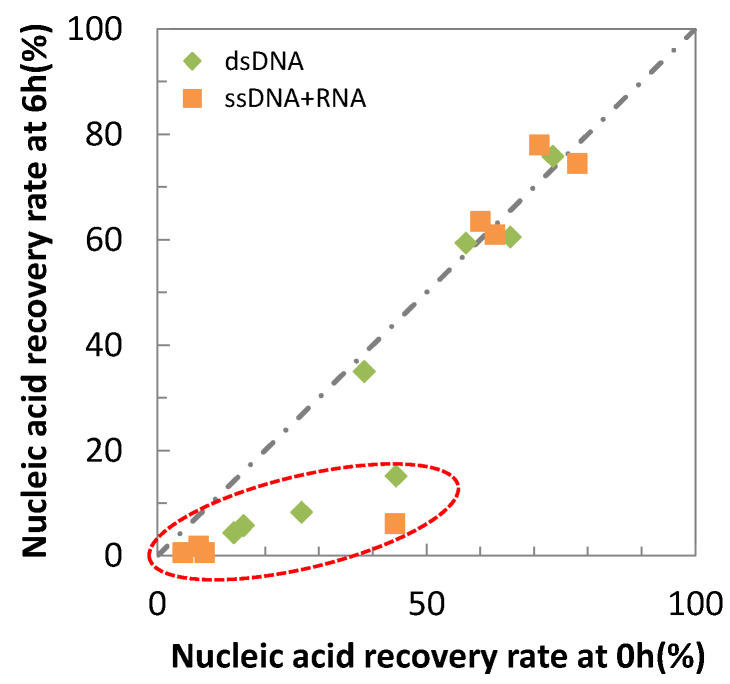
Correlation of nucleic acid recovery rates at 0 h and 6 h. The dotted line indicates nucleic acid recovery rate at 0 h equals to that at 6 h (y = x). Dashed circle indicates the results of copper and its alloys.

Figure 2 is the plot of the nucleic acid recovery rates at time “0” against those at 6 h. It clearly categorizes testing materials into two groups; materials reduced the recovery rates with the increase in the contact time, and others had constant recovery rates regardless of the contact time. The first group includes copper and its alloys.

**Figure 3:**
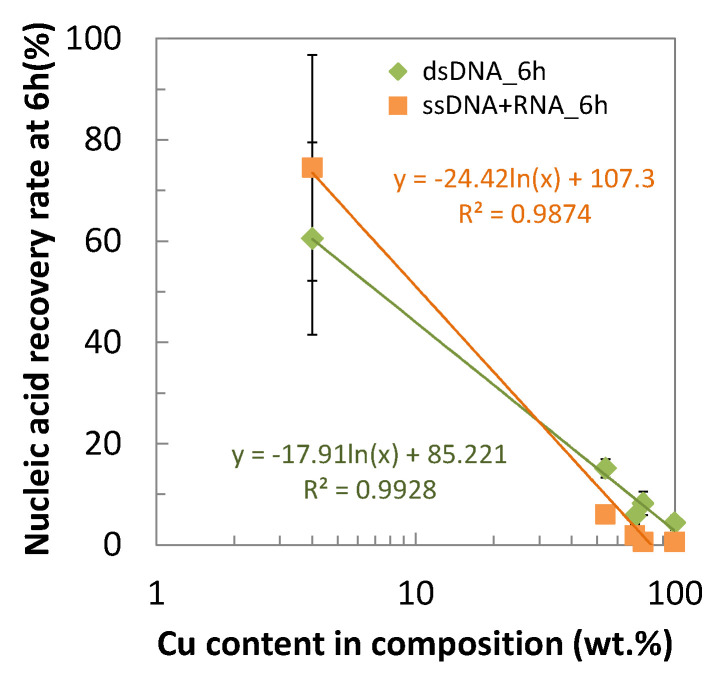
Dependence of nucleic acid recovery rate at 6 h on the copper content in the alloy composition (mean ± standard deviation).

Figure 3 plotted the nucleic acid recovery rates against the copper content in the alloy composition, indicating the alloy with the higher copper content gives the lower recovery rates, which means the higher nucleic acid degradability. It also suggests that the rapid nucleic acid degradability may be a unique property of copper and its alloys, not achieved by other commercially available antibacterial materials such as ABSS and resins.

### 2.2. Nucleic Acid Degradation by Copper Salts

**Figure 4:**
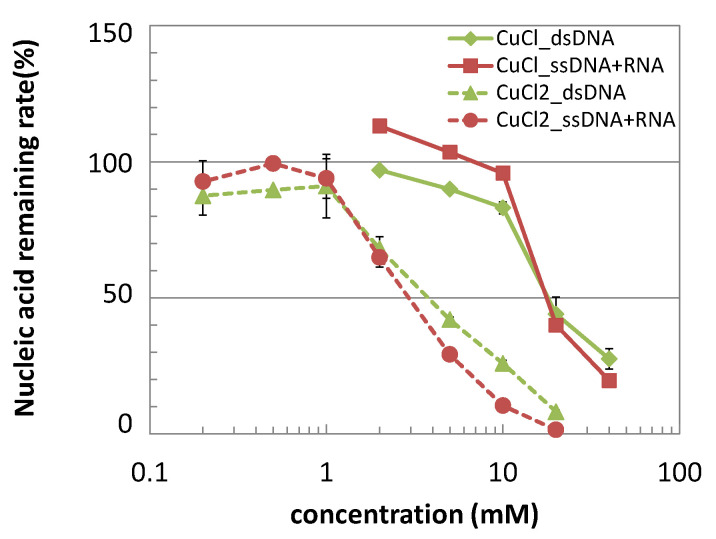
Nucleic acid remaining rates measured at 5 min after adding a certain concentration of CuCl or CuCl_2_ (mean ± standard deviation).

In regard to the antibacterial activities of the copper and its alloys, the involvement of released copper ions from the metal surface is suggested as one of the contact killing mechanisms by its uptake and accumulation into the bacterial cells [4,20]. The nucleic acid degradation by copper salts was investigated in a similar condition and shown in Figure 4. Copper (I) and (II) chlorides, which were employed as the sources of copper (I) and (II) ions, both showed the rapid degradation of nucleic acid samples within 5 min with different concentration ranges; >1 mM for copper (II) chlorides whereas >10 mM for copper (I) salts.

**Figure 5:**
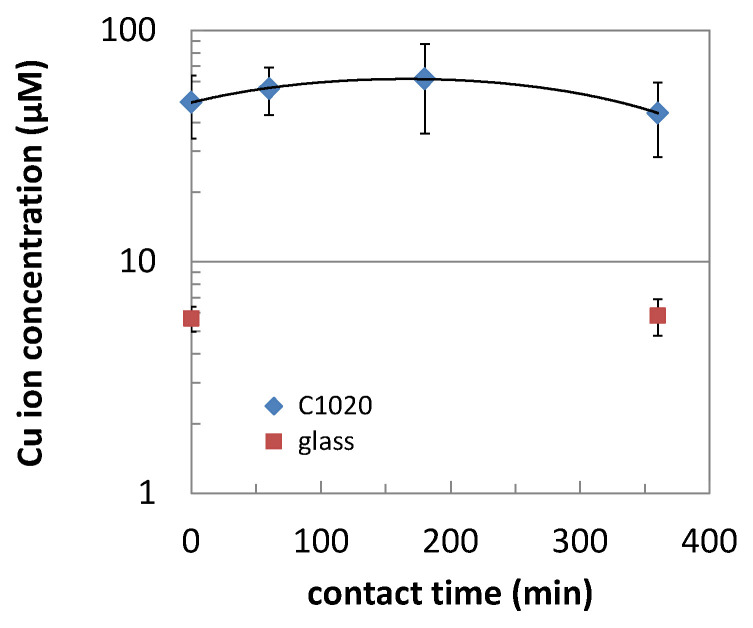
Copper ion release from C1020 under the same condition to nucleic acid degradation assay (mean ± standard deviation).

The copper ion release from the pure copper (C1020) during the nucleic acid degradation assay was analyzed by a colorimetric method, as shown in Figure 5. Since this assay was carried out under “dry” condition simulating the airborne droplets, the nucleic acid portion placed on the C1020 surface was dried within 5 min in the ambient conditions, and stayed dry during the contact time. The concentrations of the released copper ions were stable at 44–62 µM regardless of the contact time (0–6 h).

### 2.3. Antibacterial Activity of Testing Materials

**Figure 6:**
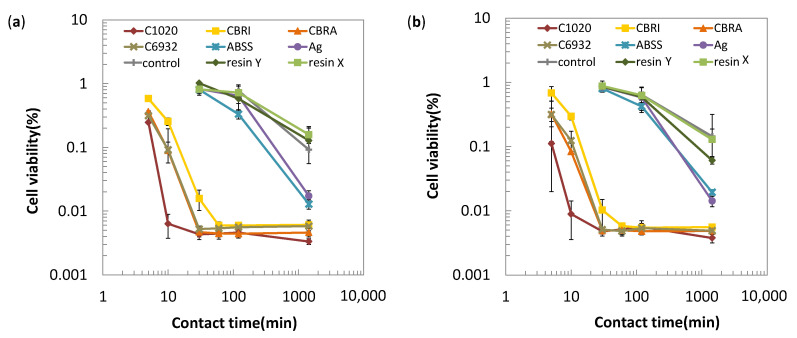
Antimicrobial activities of materials evaluated by the film method referring to JIS Z2801:2012 (ISO 22196) against *Escherichia coli* (**a**) and *Staphylococcus aureus* (**b**) (mean ± standard deviation).

In order to investigate the correlation between the nucleic acid degradability of testing materials with their antibacterial activities, antibacterial tests were carried out by a film method referring to JIS Z2801:2012 (ISO 22196) using *E. coli* and *S. aureus*. The results are shown in Figure 6 as the cell viability plotted against the contact time. Based on the results, testing materials are classified into three groups; (A) copper and its alloys, (B) silver and antibacterial stainless steel, and (C) commercially available antibacterial resins for both the Gram-negative and Gram-positive bacteria. For group A materials, the number of viable cells rapidly decreased to 1/10 of the inoculated cells within 20 min. The decreasing rate of the bacterial cells depends on the type of copper alloys, and was the largest on C1020, followed by C6932 and CLEANBRASS^®^ (CBRA), and then, by CBRI. In the case of C1020, the number of viable cells decreased to 1/100 within 10 min. For group B, the number of the viable cells decreased less than 1/10 at 24 h, but not at 30 or 120 min. For group C, the number of the viable cells was similar to that of the control material, glass after 24 h.

Based on the results in Figure 6, the time to decrease the viable cells to 1/10 or 1/100 of the inoculated number (T_1/10_ or T_1/100_) was calculated by the probit method and shown in Table 1. These parameters clearly identify the difference in the antibacterial activities of testing materials as three groups; group A with T_1/10_ of ~20 min, group B with T_1/10_ of ~500 min, and group C with T_1/10_ of >1000 min, that corresponds to the copper and the copper alloys, ABSS and Ag, and the antibacterial resins, respectively.

**Figure 7:**
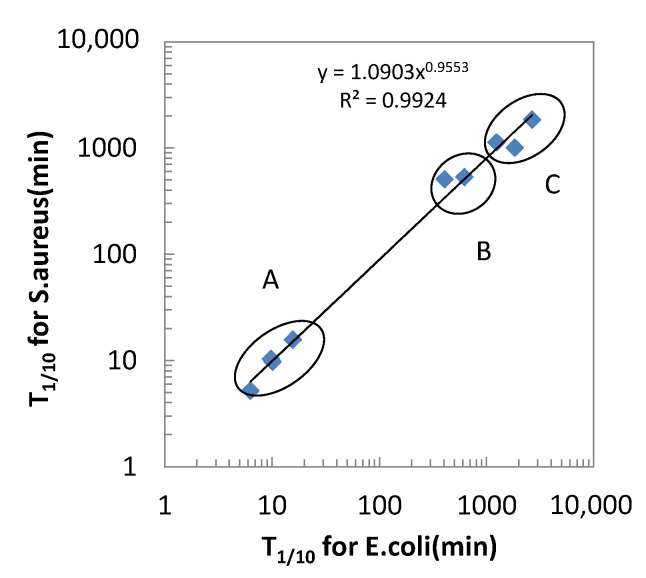
Correlation of the time to reduce the number of viable cells 1/10 of the initial number of cells (T_1/10_) for testing materials against *E. coli* and *S.aureus*. The classification of 3 groups (A–C) is identified as circled.

**Figure 8:**
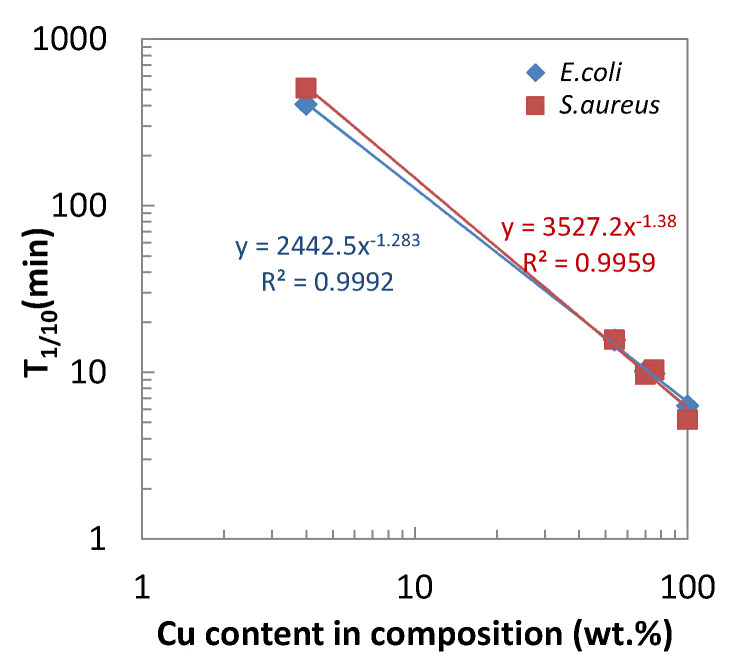
Dependence of T_1/10_ on the copper content in the alloy composition.

Figure 7 is the plot of the T_1/10_ for *E.coli* against T_1/10_ for *S.aureus*, confirming the good correlation between Gram-negative and Gram-positive bacteria. In this figure, the classification of 3 groups (A–C) is identified as circled, indicating the higher bacterial activities of group A than others. Figure 8 plotted the T_1/10_ for both kinds of bacteria against the copper content in the alloy composition. This figure suggests the higher copper content in the alloy contributes to the higher antibacterial activities.

### 2.4. Correlation between Nucleic Acid Degradability and Antibacterial Activity of Testing Materials

**Figure 9:**
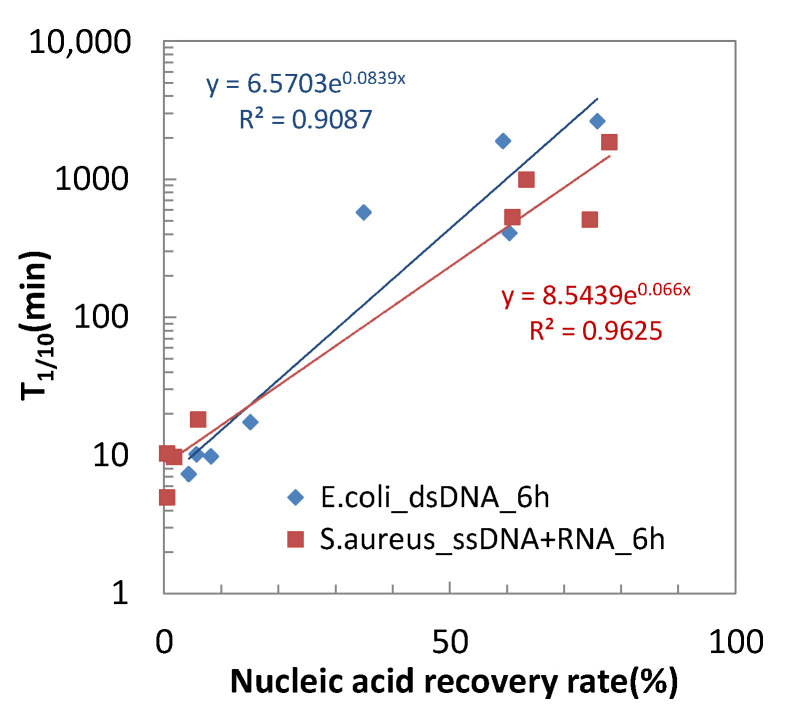
Correlation between nucleic acid recovery rate at 6 h and the antimicrobial activity, T_1/10_.

Figure 9 shows the plots of T_1/10_ for both *E.coli* and *S.aureus* against dsDNA and ssDNA + RNA recovery rates after 6 h of contact time, respectively. It clearly shows the trend that the material having low T_1/10_ has a low nucleic acid recovery rate. Even among the copper and its alloys, the higher copper content in the alloy composition resulted in the higher antibacterial activity and nucleic acid degradability.

## 3. Discussion

### 3.1. Antibacterial Activity of Copper, Copper Alloys, and Other Antibacterial Materials

Antibacterial activities of the materials were evaluated by contacting bacterial cell suspensions to the material surfaces for a certain time and counting the number of recovered cells from the testing materials. The most popular method to count the number of recovered cells is an agar plate cultivation method using 10-fold serial dilutions. This method has high sensitivity and can be performed without any special equipment, but it requires a relatively long time, such as 40–48 h, for the formation of bacterial colonies [21]. In this study, the 5-Cyano-2,3-ditolyl-2H-tetrazolium chloride (CTC) method [22,23] is employed to quantify the viable cells (with respiratory activity) under fluorescent measurement. This method has the advantage of counting the viable cells fairly quickly, such as 30 min of incubation with CTC, but it has a disadvantage of relatively low sensitivity (detection limit). Ideally, the agar plate cultivation method can detect a single cell if it can grow and form a colony, but the detection limit of the CTC method is about ~4000 cells at the condition employed in this study.

As shown in Figure 6, testing materials are classified into three groups based on their antibacterial activities, indicating the highest antibacterial activity of copper and copper alloys, followed by non-copper antibacterial metallic materials, and then, antibacterial resins. In the case of C1020, the number of viable cells decreased to 1/100 within 10 min. This rapid decrease in bacterial cells on the C1020 surface agrees with the previous reports using *Escherichia coli* (W3110 [13] and NCTC13441 [15]) and those using MRSA [18,24] even though they use slightly different cell strains with the larger inoculated number of cells. It also agreed with these reports [15,18,24] that the antibacterial activity of pure copper was higher than those of copper alloys. Generally, the higher copper content in the alloy gives the higher antibacterial activities [4,11,15,18,24,25], which is also confirmed in the present study. These facts suggest that the viable cell counts by CTC can give similar results to those by the agar plate cultivation method and that the CTC method is useful to obtain the antibacterial activities of various materials in a relatively short experimental time.

Though much research has investigated the antibacterial activity of copper and its alloys, few reports compare their antibacterial activities with those of commercially available antibacterial materials. Antibacterial stainless steel and resins employed in the present study are authenticated by the antibacterial tests following JIS Z2801:2012 [26], equivalent to ISO 22196 [21]. In this standard, antibacterial activity is defined by the comparison of the viable number of cells between the target material and that without antibacterial treatment after 24 h of contact time. The antibacterial material is required to have the recovered cells two orders of magnitude less than those from the non-treated material [26]. On this definition, it does not mean that the recovered cells from the target material are less than the inoculated cells. In the present study, the number of the bacterial cells recovered from ABSS after 24 h of contact decreased almost 1/100 of the inoculated number, suggesting the bactericidal activity on its surface. For resins X and Y, however, the numbers of the viable cells were only reduced to a similar level of the control material (glass), and not reached to 1/100 of the inoculated number. This result indicates that these resins have relatively low antibacterial activities. Unfortunately, we could not obtain detailed information about the purchased resins, but surface analysis by energy-dispersive X-ray spectroscopy (EDX) suggests the existence of the oxide of titanium and barium (Table S1). Additionally, zinc was detected on resin X, whereas a trace amount of Ag was detected on resin Y. Based on this analysis, both resins are considered to have titanium dioxide coating, which is well-known to have an antibacterial effect owing to its photocatalytic activity [27,28]. In the present study, the antibacterial assay was carried out by the film method in the safety cabinet with fluorescent light at ambient temperature, therefore, the lighting condition may not be optimal for the photocatalysis of these resins.

The antibacterial activity levels of the testing materials were indicated by the parameters T_1/10_ or T_1/100_, as shown in Table 1. The necessary antibacterial activity level of a material might differ depending on its application. For example, touch surfaces in a surgical operation room or a hospital room require relatively high levels of antimicrobial activities, whereas those in a bathroom or a kitchen of a house for a healthy person might not require the same, severe levels of it. It is reasonable to suppose that the antibacterial activity levels required for a touch surface depend on its purpose and usage environment. Therefore, the parameters such as T_1/10_ or T_1/100_ are useful information to choose appropriate materials for various touch surfaces.

### 3.2. Nucleic Acid Degradability of Copper and Copper Alloys

Though it is not fully understood, the following four mechanisms are suggested to be involved in the process of bacterial death on copper surfaces [4,20]; (1) membrane ruptures, (2) Cu^2+^ uptake and accumulation into the bacterial cell, (3) generation of reactive oxygen species, and (4) DNA degradation. Dominancy or sequences among these processes remains unclear; it may be different depending on the type of microorganisms [4]. As described before, the degradation of bacterial genes is considered as beneficial to inhibit HGT of antibiotic-resistant genes and confirmed by electrophoresis of nucleic acids extracted from bacteria applied to the copper surface [12,13,14,15,16,17,18]. However, this method has a limitation on its quantitativeness since the amount of extracted nucleic acids will be influenced by the difference in the number of viable cells recovered from the copper and the reference material. In order to avoid this influence, a nucleic acid solution is applied to the material surface and collected after a certain period of time using a swab. Since the amount of the nucleic acid solution applied is as little as 1 µL, this testing condition is closer to the actual situation in which airborne droplets contact to the touch surface.

As shown in Figure 1, the copper and its alloys have lower nucleic acid recovery rates even at time “0” (5 min) than other materials. In the case of C1020, the recovery rate is only 14.2% for dsDNA after 5 min of contact, which agrees with the result of MRSA contacting to copper, observing the disappearance of its intact DNA by staining with fluorescent intercalater [18]. The rapid decrease in the recovery rates on the copper and its alloys corresponds well to the rapid antibacterial effect of copper and its alloys described before (Figure 9). Among the copper alloys tested, CBRI had a slightly slower decrease in the recovery rates, which agrees with its slightly larger T_1/10_ or T_1/100_. This is interesting since *E.coli* death occurs ahead of genomic DNA degradation contacting C28000 (60% Cu–40% Zn alloy) [14]. In other words, genomic DNA degradation is not considered as the primary cause of “contact killing” on copper and its alloy surfaces, but their nucleic acid degradability correlates to their antibacterial activity levels. It suggests that the nucleic acid degradability measured in the present study reflects the copper surface reactivity, which causes a bactericidal effect on their surfaces. Therefore, similarly to the antibacterial activity, the nucleic acid degradability of copper and its alloys depend on the Cu content in their composition, as described in Figure 3.

Copper is known to form an oxide layer on its surface in ambient conditions by reacting with oxygen and humidity in air. Cu_2_O is initially formed, followed by the formation of CuO that is favored in a humid atmosphere with a long aging period [20]; alloying influences the composition of the naturally formed oxide layer. For example, in the case of copper–zinc alloys, the higher zinc content in the alloy increases ZnO in the surface oxide layer, suppressing Cu_2_O formation [29]. The difference in antibacterial activity of CuO and Cu_2_O is reported; thermally prepared Cu_2_O layer on the pure copper surface has higher antibacterial activity than CuO layer [30]. Ion release from the metal surface is another important point of view for the copper and its alloys antibacterial effect. Among pure copper surfaces prepared by different methods as rolling, polished, and electroplated, the latter released copper ions most, resulting in the highest antibacterial activity [31]. In the case of ABSS, heat treatment is necessary to enlarge the ε-Cu precipitates on the alloy surface, which encourages the release of copper ions [19,32]. The corrosion rates of the copper alloys in 0.9% NaCl are smaller than that of pure copper, but larger than that of ABSS (Figure S1 and Table S2), which suggests the lower copper content in the alloy composition contributes to the decrease in copper ion release, resulting in the lower antibacterial activity.

Regarding nucleic acid degradation, the involvement of released copper ions is not clear. Therefore, nucleic acid degradation by copper salts was investigated (Figure 4). It confirmed that nucleic acid degradation can occur without copper or its alloy surface though their concentrations are relatively high as >1 mM for CuCl_2_ and >10 mM for CuCl. The copper ion release from the C1020 in the same condition to the nucleic acid degradation assay was 44–62 µM in 20 µL (Figure 5), which was over 100 times smaller than those of the copper salts causing the nucleic acid degradation. However, the concentrations of copper ions released were a similar level in spite of different contact times (0–6 h) in a dry condition, suggesting that the copper ion release mainly occurred during the drying process of the nucleic acid solution, i.e., first 5 min, or during the swabbing process (~30 s). For simplification, we suppose the copper ion release occurred during the first 5 min, resulting in the copper ion concentration in the 1 µL portion of the nucleic acid solution as 0.88–1.23 mM. The drying process reduces the amount of nucleic acid solution to less than 1 µL, therefore, the copper ion concentration might be higher than this value. This hypothetic calculation suggests the possibility that the released copper ions may be responsible for the nucleic acid degradation on the copper surface. In the research using MRSA, the involvement of Cu^+^ and Cu^2+^ into genomic DNA fragmentation was confirmed by the addition of chelating reagents to the testing media [18], which coincides with the result in the present study.

Generally, the fragmentation of the bacterial gene applied to the copper surface is assigned to the generation of relative oxygen species by the Fenton-like reaction [4] shown as the following equation.

Cu^+^ + H_2_O_2_ → ⋅OH + OH^−^ + Cu^2+^

This requires H_2_O_2_, which is not included in our experimental condition. In our nucleic acid degradation assay, nucleic acid degradation may be caused by a different mechanism. In an aqueous solution, the corrosion reaction of the copper is described in the following equation [33];

2Cu + O_2_ + 2H_2_O→ 2Cu^2+^ + 4OH^−^

where the Cu releases 2 electrons to oxygen, resulting in the generation of OH^−^. The generation of hydroxyl ions may contribute to the fragmentation of RNA since it is easily hydrolyzed under alkaline conditions by the nucleophilic attack from the 2-hydroxyl group of ribose to the phosphodiester bond [34]. The affinity of divalent cations to the nucleobases were estimated [35] and copper ion had a relatively high affinity constant, suggesting the possibility of chelating to the nucleobases in the single or double stranded nucleic acids. Coordination of metal ions to the nucleobases may influence the conformation of nucleic acids [35], which may enable nucleophilic attack on the phosphodiester bond from other hydroxyl groups. Under the drying process of 1 µL of nucleic acid solution on the copper surface, a relatively high concentration of copper ions, as well as a pH increase, may occur, which may accelerate the degradation of the nucleic acids. The higher release of copper ions, that is, the higher rate of corrosion, most likely results in the higher copper ion concentration and the higher pH, which can accelerate more the nucleic acid degradation. Further investigation is necessary to elucidate the mechanism of nucleic acid degradation on copper and its alloy surface.

Recently, the degradation of viral nucleic acids on copper surface is reported [36,37], as well as denature of capsid proteins of human norovirus-like particles [38]. These phenomena are also considered as attributable to the high reactivity of copper and its alloy surface, which we can quantify as the nucleic acid degradability using this developed method. The usefulness of this method is to simulate/estimate the antibacterial/antiviral activity of materials under the situation simulating airborne droplet contact. No biosafety requirement is applied to this nucleic acid degradation assay, therefore, you can perform this assay on the touch surface in the real world, such as in hospital rooms. This is one of the advantages of this assay and it can offer a useful measure of the antibacterial/antiviral activity in a real environment.

## 4. Materials and Methods

### 4.1. A Testing Materials

Materials used in this study are oxygen-free copper (C1020), three kinds of copper alloys (C6932, CLEANBRIGHT^®^ abbreviated as CBRI, and CLEANBRASS^®^ abbreviated as CBRA), antibacterial stainless steel (NSSAM3, abbreviated as ABSS), pure silver (>99.99%), and two kinds of antibacterial resins (X and Y). All copper and copper alloys are supplied by Mitsubishi Materials Corporation. Antibacterial stainless steel, silver, and two kinds of resins are commercially available. Chemical compositions of oxygen-free copper and other alloys are shown in Table 2.

All metal samples wre cut into 15–20 mm squares and 0.5–2 mm thick for this antibacterial assay. Specimens of antibacterial resins were cut from the purchased toilet seats and lids into 15 mm square and 3–5 mm thick. Metal specimens were ground by SiC paper upto #1200 (~5 µm), followed by rinsing with ultrapure water. Resin specimens were cleaned by wiping with absolute ethanol.

For nucleic acid degradation assay, metal specimens of 15 cm square and 0.5–2 mm thick were prepared in the same manner except for C6932, which was prepared in disks of 60 mm in diameter and 4 mm thick. For resins, ~15 mm square and 3–5 mm thick specimens were prepared in the same manner as the antibacterial assay. The specimen surfaces were cleaned by a commercially available detergent (a mixed solution of sodium α-dodecan-1-yl-ω-(sulfonatooxy)poly(oxyethylene) and fatty acid alkanolamide) and thoroughly rinsed by ultrapure water prior to the assay.

### 4.2. Nucleic Acid Degradation Assay

**Figure 10:**
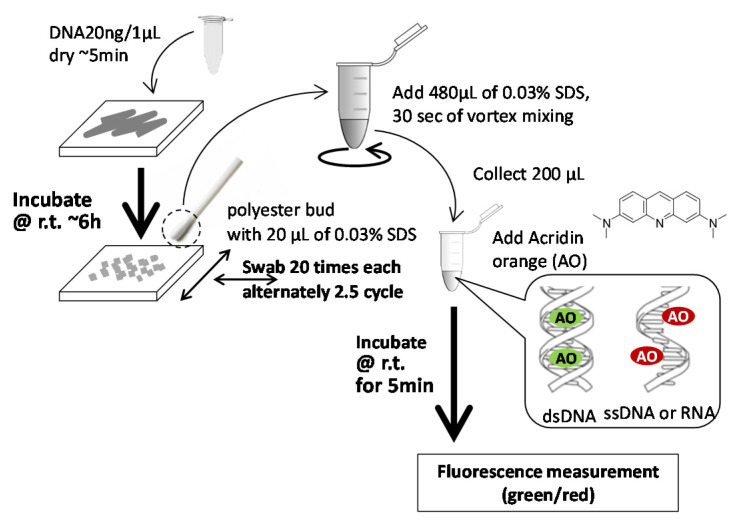
Schematic explanation of the procedure for the evaluation of the nucleic acid degradability of material surfaces by a swab method.

The schematic explanation of the assay procedure for nucleic acid degradation on the material surface is shown in Figure 10. Deoxyribonucleic acid (DNA) from salmon sperm for molecular biology (FUJIFILM Wako Pure Chemical Corporation, Osaka, Japan) was used as a nucleic acid source. A 1 µL portion of 20 mg/mL DNA in 0.5% polyoxyethylene(10) octylphenyl ether (Triton X-100) solution was spread over the area about 10 mm × 10 mm on the testing material surface using a tip of a digital pipette. In the case of 15 cm samples, it was divided into four areas and one portion was loaded into each area. The surfactant was added to be easily spread the DNA solution over the sample surface. It takes ~5 min to dry the DNA portion completely. This time point is described as “0”. DNA-loaded samples were incubated at room temperature for 1, 3, and 6 h, and then, DNA portion was collected by a polyester swab with 20 µL of 0.03% sodium dodecyl sulphate (SDS). The swab was applied 20 times horizontally and 20 times vertically, alternately up to 5 sets (100 times in total) covering the DNA loaded area. The head of the swab was cut into a 480 µL portion of 0.03% SDS and vortexed for 30 sec. to extract collected DNA. Then, DNA in SDS was quantified by the acridine orange (AO) method with a calibration curve prepared using the dilutions of DNA solution with 0.03% SDS. A 1.2 µL portion of 0.5 mg/mL AO solution (stored at 0 °C) was added to a 200 µL portion of the DNA extract with SDS. AO is known to emit green (λ_Ex_ = 502 nm, λ_Em_ = 526 nm) fluorescence by binding to dsDNA whereas it emits red (λ_Ex_ = 460 nm, λ_Em_ = 650 nm) fluorescence by binding to ssDNA or RNA. Therefore, the fluorescence of the solution was measured at excitation by a blue LED (∼470nm) and emission through a green (510–580 nm) or red (665–720 nm) filter using the benchtop fluorometer (Qubit 3.0 Fluorometer, Thermo Fisher Scientific KK, Tokyo, Japan) after 5 min at room temperature (22 ± 1 °C). The DNA extract was diluted with 0.03% SDS, if necessary. Obtained data of AO fluorescence was converted to the concentration of dsDNA or ssDNA + RNA based on the calibration curves prepared using the dilutions of the DNA source. Experiments were performed in triplicate. Prior to the material evaluation, the assay condition was optimized at the following points; (1) concentrations of loading DNA and Triton X-100, (2) a type of swabbing bud (polyester, not polyurethane or cotton), (3) a swabbing protocol (no DNA was collected after 20 × 5 times swabbing from glass surface loaded by DNA), and (4) concentrations of AO and SDS for nucleic acid quantification.

The nucleic acid degradability of CuCl_2_ and CuCl was examined in a similar manner using their suspension in 0.03% SDS. A 90 µL portion of 22.3 mg/mL DNA in 0.5% Triton X-100 solution was mixed with 10 µL of CuCl_2_ or CuCl solution appropriately diluted with 0.03% SDS. After 5 min, this mixture of DNA with copper salt was diluted 100 times with 0.03% SDS, and then, a 10 µL portion was mixed with 190 µL of 0.03% SDS and 1.2 µL of 0.5 mg/mL AO solution. After 5 min, the fluorescence of the supernatant was measured at excitation by a blue LED and emission through a green or red filter. If necessary, the DNA extract was diluted with 0.03% SDS. Experiments were performed in triplicate.

### 4.3. Measurement of Copper Ion Release

Copper ion release from C1020 during the nucleic acid degradation assay was measured by the following procedure. A 1 µL portion of 0.5% Triton X-100 was spread in the same manner to the nucleic acid degradation assay on the C1020 specimen surface. It takes ~5 min to dry completely, which is the time point of “0”. The specimens were incubated at room temperature for 1, 3, and 6 h, and then its surface was swabbed in the same manner as the nucleic acid degradation assay. The head part of the swab was cut into a 475 µL portion of 0.03% SDS and mixed (shaken) for 30 sec. to extract copper ions. Then, copper ions in this extract were quantified using the Metallo Assay Copper Low Concentrate Assay Kit LS (Metallogenics Co. Ltd., Chiba, Japan) following its instruction supplied with the kit. Briefly, a 396 µL portion of the extract was added to 4 µL 1 M hydrochloric acid (HCl) to adjust its pH as 2–3. Then, a 100 µL portion was added to 140 µL of the mixture of the buffer and chelate color reagent. After 10 min at room temperature, absorbance at 590 nm was measured by a microplate reader (Multiskan FC, Thermo Fischer Scientific KK, Tokyo, Japan). Obtained absorbance was converted to the concentration of copper, based on a calibration curve prepared using the dilutions of copper standard solution. Experiments were performed in triplicate.

### 4.4. Antibacterial Assay

The bacterial cell lines used were *Escherichia coli* (ATCC 8739, EZ-PEC^TM^, Microbiologics^®^, purchased from EnBio Ltd., Tokyo, Japan) and *Staphylococcus aureus* (ATCC 6538, EZ-PEC^TM^, Microbiologics^®^, purchased from EnBio Ltd., Tokyo, Japan). The former was employed as a representative of Gram-negative bacteria, whereas the latter was a Gram-positive. Each of the testing materials was placed on the bottom of the glass dish separately. A 50 µL portion of cell suspension containing ~1 × 10^6^ cells in 0.9% NaCl were placed onto a testing material and covered by a polyethylene film of 10 mm square to control the contacting area of the bacterial suspension. As a control surface, bacterial cells were loaded to the bottom of a glass dish in the same manner. Then, the samples were incubated at 35 ± 1 °C and relative humidity over 90% for 5, 10, 30, 60, 120, or 1440 min. After incubation, 1 mL of the mixture of 0.9% NaCl and 0.1 mM ethylenediamine-*N*,*N*,*N’*,*N’*-tetraacetic acid, disodium salt, dihydrate (EDTA-2 Na) was poured and pipetted three times over the sample in the dish to collect survived bacterial cells. Then, a 200 µL portion was used for a viable cell count using 5-cyano-2,3-ditolyl-2H-tetrazolium chloride (CTC) staining kit (-Bacstain- CTC Rapid Staining Kit for Flow cytometry, BS01, Dojindo Laboratories, Kumamoto, Japan) following its instruction. Briefly, 4.2 µL of the mixture of CTC and enhancer reagent was added. After incubation for 30 min at 35 ± 1 °C, fluorescence was measured at excitation through blue (430–495 nm) filter and emission through red (665–720 nm) filter using a benchtop fluorometer. Obtained data of CTC fluorescence was converted to the number of cells based on the calibration curves prepared using the dilutions of bacterial cell suspension. All the experiments were performed at least in duplicate and in triplicate where necessary.

## 5. Conclusions

In the present study, nucleic acid degradability of Cu and its alloy surfaces was quantitatively evaluated by a new in vitro method and compared to those of commercially available antibacterial resins, antibacterial stainless steel, and pure Ag. As a result, only Cu and its alloys showed continuous degradation of nucleic acids up to 6h of contact time, which may be attributed to the copper ion release from the Cu and its alloy surfaces. The nucleic acid degradability evaluated in this new method has a good correlation to the antibacterial levels evaluated by a film method. The higher Cu content in the alloy composition results in the higher nucleic acid degradability and the higher antibacterial activities.

## Figures and Tables

**Table 1:** Time to reduce the number of viable cells to 1/10 and 1/100 of the inoculated number of cells on the material surface (T_1/10_ and T_1/100_, min).

		C1020	C6932	CBRA	CBRI	ABSS	Ag	Glass	Resin X	Resin Y
*E.coli*	T_1/10_	6.27	9.97	10.2	15.7	383	575	1230	1830	2670
	T_1/100_	9.33	24.6	23.4	34.6	1490	1990	4060	10,500	20,400
*S.aureus*	T_1/10_	5.30	10.3	9.89	15.8	504	530	1120	1000	1850
	T_1/100_	9.86	25.0	23.8	30.7	2030	1780	5280	4630	10,900

Antimicrobial activity was evaluated by the film method referring to JIS Z2801:2012 (ISO 22196) except culture media (0.9%NaCl) and extract solution (0.9% NaCl + 0.1 mM EDTA-1Na). T_1/10_ and T_1/100_ were calculated by the probit method. CBRA: CLEANBRASS^®^, CBRI: CLEANBRIGHT^®^, ABSS: antibacterial stainless steel [19], resins X and Y were cut from the commercially available antimicrobial toilet seats and lids.

**Table 2:** Chemical compositions of oxygen free copper and alloy samples (wt.%).

Sample	Cu	Zn	Sn	Ni	Mn	Cr	Fe	Si	P
C1020	>99.99								
C6932	75.4	Rem.						3.1	0.09
CBRA	70.1	Rem.	0.5	2.0					
CBRI	54.0	Rem.	0.4	10.9					
ABSS	3.8			9.4	1.4	18.1	Rem.	0.6	

## Data Availability

The data presented in this study are available on request from the corresponding author.

## Supplementary Materials

The following are available online at https://www.mdpi.com/article/10.3390/antibiotics10121439/s1, Figure S1: Potentiodynamic curves of copper and copper-containing alloys in 0.9% NaCl, Table S1: Results of surface analysis of commercially available antibacterial resins (wt%), and Table S2: Corrosion parameters of copper and its alloys in 0.9% NaCl.
