# Supplementary material for: Quantitative Evaluation of Nucleic Acid Degradability of Copper Alloy Surfaces and Its Correlation to Antibacterial Activity
Source: Antibiotics (Basel). 2021 Nov 24;10(12):1439. doi: 10.3390/antibiotics10121439 (PMC8698190; doi:10.3390/antibiotics10121439)
Supplement: Supplementary file 1 [file antibiotics-10-01439-s001.zip › antibiotics-1457295-supplementary.pdf]

Table S1. Results of surface analysis of commercially available antibacterial resins (wt%).

element	Resin X	Resin X
C	93.51±0.34	97.63±1.24
O	3.28±0.26	0.66±1.32
Ti	0.96±0.09	0.54±0.02
Zn	1.17±0.25	-
Ba	1.23±0.14	0.71±0.07
Mg	-	0.10±0.01
Na	-	0.17±0.01
Ag	-	0.03±0.03
Al	0.11±0.03	0.09±0.01
Si	0.05±0.02	0.05±0.01

Antimicrobial resin specimens were prepared from the commercially available toilet seats and lids. Surface analysis was carried out using a scanning electron microscope (SEM, Hitachi SU-8000) equipped by the energy dispersive X-ray spectroscopy (EDX, Bruker Quantax 70).

Table S2. Corrosion parameters of copper and its alloys in 0.9% NaCl.

	C1020	C6932	CBRA	CBRI	ABSS
E_{corr} vs NHE(mV)	62.8±9.8	57.8±12.1	71.8±7.1	45.6±9.2	138.0±9.4
I_{corr}($\mu A/cm^2$)	2.65±1.00	0.42±0.32	0.29±0.15	0.70±0.15	0.052±0.001
E_{pitt} vs NHE(mV)	-	-	-	-	582±9

Electrochemical measurement condition was as follows;
Reference electrode: saturated Ag/AgCl (3 M NaCl)
Counter electrode: a platinum mesh
Working electrode: a test sample (exposed area of 0.95 cm^2)
Environment: 5 ml 0.9% NaCl, 35±1˚C.
Conditions: OCP for 24 h, PD at 1 mV/s from −0.25 V vs. OCP to the current density over 1×10^{-3} A/cm^2
Potentiostat: ALS Model2323 bi-potentiostat
Number of measurement: >3

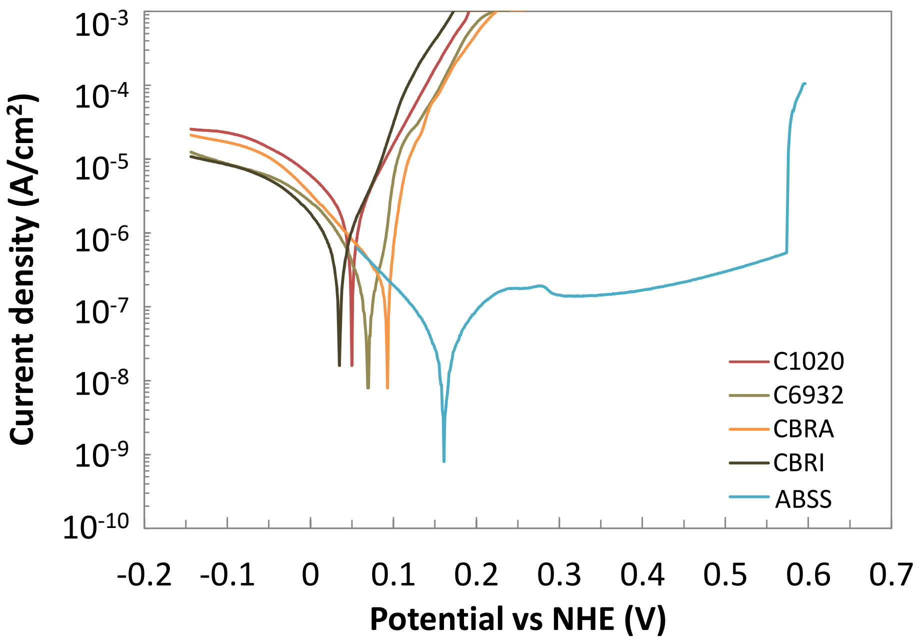

Figure S1. Potentiodynamic curves of copper and copper containing alloys in 0.9% NaCl. (Details of measuring conditions are described in the footnote of Table S2.)
